# Deciphering Thymic Development

**DOI:** 10.3389/fimmu.2014.00424

**Published:** 2014-10-08

**Authors:** Harald Von Boehmer

**Affiliations:** ^1^Department of Microbiology and Immunobiology, Harvard Medical School, Boston, MA, USA; ^2^Department of Cancer Immunology and AIDS, Dana-Farber Cancer Institute, Boston, MA, USA

**Keywords:** MHC-restricted antigen recognition, T cell clones, TCR alpha/beta cDNA clones, HY antigen, TCR transgenic mice, negative thymic selection, positive thymic selection

In 1979, some of us were surprised by the (1) conclusion of Doherty and Zinkernagel on MHC-restricted antigen recognition following the lead of Katz, Hamaoka, and Benacerraf (2) describing the same for the interaction of T helper cells with B cells as well as Rosenthal and Shevach (3) describing it for the interaction of T cells with macrophages. Since Doherty and Zinkernagel offered the least complicated system, they got most of the credit. We wanted to know whether a single effector cell was involved and therefore analyzed a clone of cells, specific for the HY transplantation antigen. If it could be shown that the progeny of a single cell was MHC restricted, we had something to explain, which was not obvious. The cloning in Basel worked fine, with the competent help of Hans Hengartner before his departure to the lab of Zinkernagel in Zurich. The clone was in fact MHC restricted, telling us that MHC restriction was the property of a single cell. The same conclusion was derived from experiments with Matthias Wabl, who observed the killing of targets by single killer cell (4). The clone was also alloreactive, which was observed prior to the realization that a significant portion of T cells carried two receptors and thus it is unclear to date whether a second receptor was involved or not. This was just the first example of a clone, which was MHC restricted and HY specific as well as H-2Dd specific and this overlap in specificity was subsequently observed in other clones such that the high frequency of alloreactive T cells is not really an issue.

Follow up experiments with the Michael Steinmetz lab transferring TCR alfa and beta genes from one T cell clone to another allowed us to unequivocally conclude that the MHC-restricted specificity was encoded by a single receptor long before crystallographic studies reached the same conclusion (5). This surprised some molecular biologists somewhat who thought that the cloning of the TCR put an end to the mysteries of the immune system.

This lead automatically to the next step, the construction of TCR transgenic mice, to analyze the selection of T cells according to their specificity. Initially, we were interested to test the ideas of Burnet and Lederberg that autoaggressive cells were eliminated in primary lymphoid organs. For this reason, we used again the genes of HY specific clones since then we could easily compare female and male mice. Here, I have to tell a little tale that characterizes (some?) scientists: it was Michael Steinmetz, who had previously spoken to Fritz Melchers, who asked at the Reisensburg in the South of Germany whether there would be any interest in generating TCR transgenic mice. I answered with a clear yes saying that this would allow to test Burnets and Lederbergs ideas. So, it was concluded to go ahead and initially Georges Koehler was singled out as the scientist residing by now in Freiburg to help with the construction of mice since he had succeeded to generate immunoglobulin transgenic mice. I was therefore mildly surprised when one day Georges entered my lab and asked me whether I could give him an HY specific clone since he had the idea of testing Burnets and Lederbergs ideas. I told him that this sounded familiar, he blushed only a little and then asked Hans Georg Rammensee who was in the same office a related question. I leave it to the audience to imagine what Michael Steinmetz told Georges Koehler or better what he did not tell him, even though one cannot be completely sure of it. So Georges did not produce the mice but Anton Berns in Amsterdam cooperated and very nicely mapped what was required to express TCR beta genes in transgenic mice. Finally, the co-injection of alfa and beta genes from an HY specific clone was done by Horst Bluethmann at Hoffmann La Roche in Basel where Michael Steinmetz had moved. When the mice had grown up, we tested them with a variety of reagents prepared for this task and could report on the deletion of CD4^+^8^+^ cells in male mice even though these mice came with an anomaly, the too early expression of the transgenic TCR, which made proper quantitation difficult (6). Only recently could we address this problem and reported deletion of CD4^+^8^+^ thymocytes in the absence of TCR editing (7). This ended a long story on the deletion of autoaggressive cells at a certain stage of development, something that had not been addressed in mice expressing superantigen specific receptors, which somewhat compromised our transgenic approach since they were conducted later and yielded results earlier albeit with the limitation that the conclusions had to be restricted to superantigens (8) whereas we dealt with conventional antigens for T cells.

The next step was related to positive selection and the matching of specificity and function. Here, the first realization was that a receptor derived from a CD8^+^ cell would only be expressed on CD8^+^ cells in the transgenic mice (9, 10). The second was that there was in fact positive selection as mice with inappropriate MHC antigens not restricting the specificity of the cell from which receptor genes were obtained, failed to generate single positive cells and thus development was arrested at the CD4^+^8^+^ stage where cells died (9). This was then named death from neglect as opposed to death by negative selection which eliminated likewise CD4^+^8^+^ cells, at least when the receptor was derived from CD8^+^ cells (7). It was then clear that it was the MHC molecules expressed in the thymus and the TCR specificity, which determined positive selection, which also led to the matching of specificity and function (10), such that CD8^+^ killer cells were generated from immature cells expressing a class I restricted TCR (11) and as shown later CD4^+^ helper cells were generated from immature cells expressing a class II restricted TCR and thus in other words helper cells recognized as a rule peptides entering the target cell from the outside whereas killer cells recognized peptides produced in the target cell itself. This relates to the different modes of peptide loading by class I and class II MHC antigens (12–16).

In the meantime, the molecular details of this matching process have been worked out mostly by the work of Dietmar Kappes (17) as well as Dan Littman (18) who identified transcription factors guiding this process in dependence of the signaling by the receptor expressed by immature cells. Thus, at present we have a fairly complete picture of positive selection as far as the selectable T cells are concerned while still we know relatively little about the TCR ligands that are responsible for positive selection. Here, one wonders whether thymus-specific proteasome subunits play an essential role (19). Thus, there are still some secrets in T cell development even after decades of the identification of the TCR (20).

The curiosity in T cell development is still very much alive even after retirement but I trust that the remaining issues are in good hands of younger scientific colleagues who identify the outstanding questions and think of clever experiments to address them.

## Conflict of Interest Statement

The author declares that the research was conducted in the absence of any commercial or financial relationships that could be construed as a potential conflict of interest.
